# Age-related differences of subjective visual vertical perception in adults—a functional near-infrared spectroscopy study

**DOI:** 10.3389/fnagi.2024.1449455

**Published:** 2025-01-07

**Authors:** Jun Lu, Xiang Gong, Meng-Huan Wang, Ruo-Xin Zhao, Yu-Chen Wang, Ying-Ying Shen, Rong Cao, Guang-Xu Xu

**Affiliations:** ^1^Department of Rehabilitation Medicine Center, The First Affiliated Hospital of Nanjing Medical University, Nanjing, Jiangsu, China; ^2^School of Rehabilitation Medicine, Nanjing Medical University, Nanjing, Jiangsu, China; ^3^School of Chinese Language and Literature, Nanjing Normal University, Nanjing, Jiangsu, China; ^4^Office of Clinical Trial Institution, School of Medicine, Nanjing Zhongda Hospital, Southeast University, Nanjing, Jiangsu, China; ^5^Department of Health Promotion Center, The First Affiliated Hospital with Nanjing Medical University, Nanjing, Jiangsu, China; ^6^School of Sport and Health, Guangzhou Sport University, Guangzhou, Guangdong, China

**Keywords:** subjective visual vertical, vertical perception, functional near-infrared spectroscopy, cortical activation, brain functional lateralization

## Abstract

**Background:**

The perception of Subjective Visual Vertical (SVV) is crucial for postural orientation and significantly reflects an individual’s postural control ability, relying on vestibular, visual, and somatic sensory inputs to assess the Earth’s gravity line. The neural mechanisms and aging effects on SVV perception, however, remain unclear.

**Objective:**

This study seeks to examine aging-related changes in SVV perception and uncover its neurological underpinnings through functional near-infrared spectroscopy (fNIRS).

**Methods:**

In a comparative study of 19 young and 19 older adults, the standardized SVV task executed in Eprime 3.0 software evaluated participants’ SVV orientation and uncertainty. Cortical responses were monitored via fNIRS during the task, with block averaging analysis employed to delineate the associated hemodynamic responses. The study further correlated these neuroimaging findings with behavioral measures.

**Results:**

Young individuals exhibit superior accuracy and stability in perceiving the subjective visual vertical (SVV) direction. Neuroimaging data, adjusted for multiple comparisons using the false discovery rate, reveal activation of the right supramarginal gyrus (SMG) and the left dorsolateral superior frontal gyrus (SFGdor) in both age groups during SVV tasks. However, older participants show additional activation in regions such as the bilateral postcentral gyrus (PoCG) and the right middle frontal gyrus (MFG). Lateralization studies indicate that young participants predominantly exhibit right lateralization in sensory and dorsolateral prefrontal cortices, with left lateralization in the motor cortex. In contrast, elderly participants demonstrate bilateral dominance across sensory, dorsolateral prefrontal, and motor cortices. Correlational analyses link modified SVV metrics to the activation levels of various brain regions, with negative correlations observed in both age groups, and a unique positive correlation with the left inferior frontal gyrus of the triangular part (IFGtriang) in young participants.

**Conclusion:**

Young individuals outperform the older individuals in SVV performance due to age-related differences in brain functional patterns during the execution of vertical perception judgment. Both age groups activate the right SMG and left SFGdor, but the older individuals additionally activate regions such as bilateral PoCG and right MFG. While young people exhibit right-brain dominance, the older people rely on bilateral cognitive resources, indicating bilateral dominance. Except for the left IFGtriang in the young, higher activation in brain regions correlates with better SVV performance.

## 1 Introduction

The perception of verticality relates to a subject’s ability to determine an Earth vertical line without external reference cues (Pawlitzki et al., 2018) and is a critical component of normal balance and gait (Yi et al., 2007; Perennou et al., 2008; Dai et al., 2021). Vertical perception has been demonstrated to vary between young and older individuals (Cakrt et al., 2016). And directional deviations increase with age, regardless of gender (Gerb et al., 2023). One possible explanation is that aging may have an impact on the vestibular system, leading to sensory reweighting of information. SVV is an important indicator of the condition of vestibular function. When related diseases cause damage to normal vestibular function, the function of SVV will become abnormal, and its value will exceed the normal range (Baier et al., 2022; Bogle et al., 2022; Choi et al., 2022). With age, bilateral vestibular hypofunction is often progressive and can result in balance and spatial orientation problems (Alberts et al., 2019). In the older population, perception and gravity are closely related to action and function (Manckoundia et al., 2007). Variability of vertical perception has been proven to increase the risk of falls in older individuals and is a predictor of falling occurrences (Menant et al., 2012). The input of sensory information changes with age in the judgment weight of vertical perception, and it has been pointed out that the weight of vestibular information decreases with age, while the weight of visual information increases (Alberts et al., 2019). Using the SVV assessment indicator, understanding the vestibular dysfunction across different age groups is of great significance for maintaining upright balance and preventing falls in the older people.

Vertical perception ability is usually evaluated using three subjective judgment methods: subjective visual vertical (SVV), subjective postural vertical (SPV), and subjective haptic vertical (SHV). Among these, SVV has gained recognition and widespread adoption among researchers due to its paradigm stability and versatility in research applications (Piscicelli et al., 2015). The SVV task mainly refers to vertical perception judgment by integrating visual and vestibular system information (Piscicelli et al., 2015; Piscicelli and Perennou, 2017), and it can detect the dysfunction of the peripheral or central vestibular system (Dieterich and Brandt, 1993). The previous research has acknowledged the significant involvement of specific brain regions, namely the parietal insula cortex, posterior lateral thalamus, and insula, in constructing the internal perceptual models for vertical perception (Lemaire et al., 2021). In studies of stroke patients, it has been shown that damage to the relevant brain areas can lead to impairments in vertical perception functions (Colebatch et al., 2016). In particular, it can lead to differences in the function and functional connectivity of the cortical regions involved in visual vertical perception in the vestibular cortex (Jafari et al., 2023). According to Kheradmand et al. (2015) and Babyar et al. (2019), the right supramarginal gyrus (SMG) plays an important role in the process of vertical perceptual judgments at the cortical level.

However, there is a lack of direct evidence of the involvement of the relevant regions in the execution of vertical perceptual functions.

Functional near-infrared spectroscopy (fNIRS), a cutting-edge non-invasive optical imaging system, has demonstrated its great utility in indirectly assessing neuronal activity by quantifying oxyhemoglobin (HbO) and deoxyhemoglobin (HbR) levels within tissues. Rigorous scientific investigations have substantiated its reliability and validity considerably (Chen et al., 2020). fNIRS has emerged as an indispensable tool for elucidating the neural mechanisms underlying spatial perception. It enables researchers to investigate the dynamic activity patterns within cortical brain regions during the execution of intricate visuospatial tasks, encompassing spatial coding and visuospatial perception. These pioneering studies employing fNIRS have significantly advanced our comprehension of the complex neural mechanisms involved in spatial cognition (Haberstumpf et al., 2020; Derbie et al., 2021). However, to date, no research has yet applied relevant functional imaging devices to investigate cortical activation in individuals during the execution of the SVV task. Compared to fMRI, fNIRS exhibits greater tolerance towards motion artifacts and does not impose strict requirements on participants’ posture during task execution (Pinti et al., 2020). Thus, in contrast to traditional MRI procedures where subjects typically lie down, fNIRS offers a distinct advantage for SVV paradigms that necessitate a seated position. This study aims to scrutinize brain region activation patterns during subjective vertical perception judgment tasks with the primary goal of establishing a foundation for improving the internal model of vertical perception. Additionally, the experiment seeks to find the relationship between brain image and behavior and identify potential targets for non-invasive brain modulation interventions, such as transcranial magnetic stimulation, in addressing postural control disorders associated with vertical perception dysfunction.

## 2 Materials and methods

### 2.1 Participants

**Figure 1:**
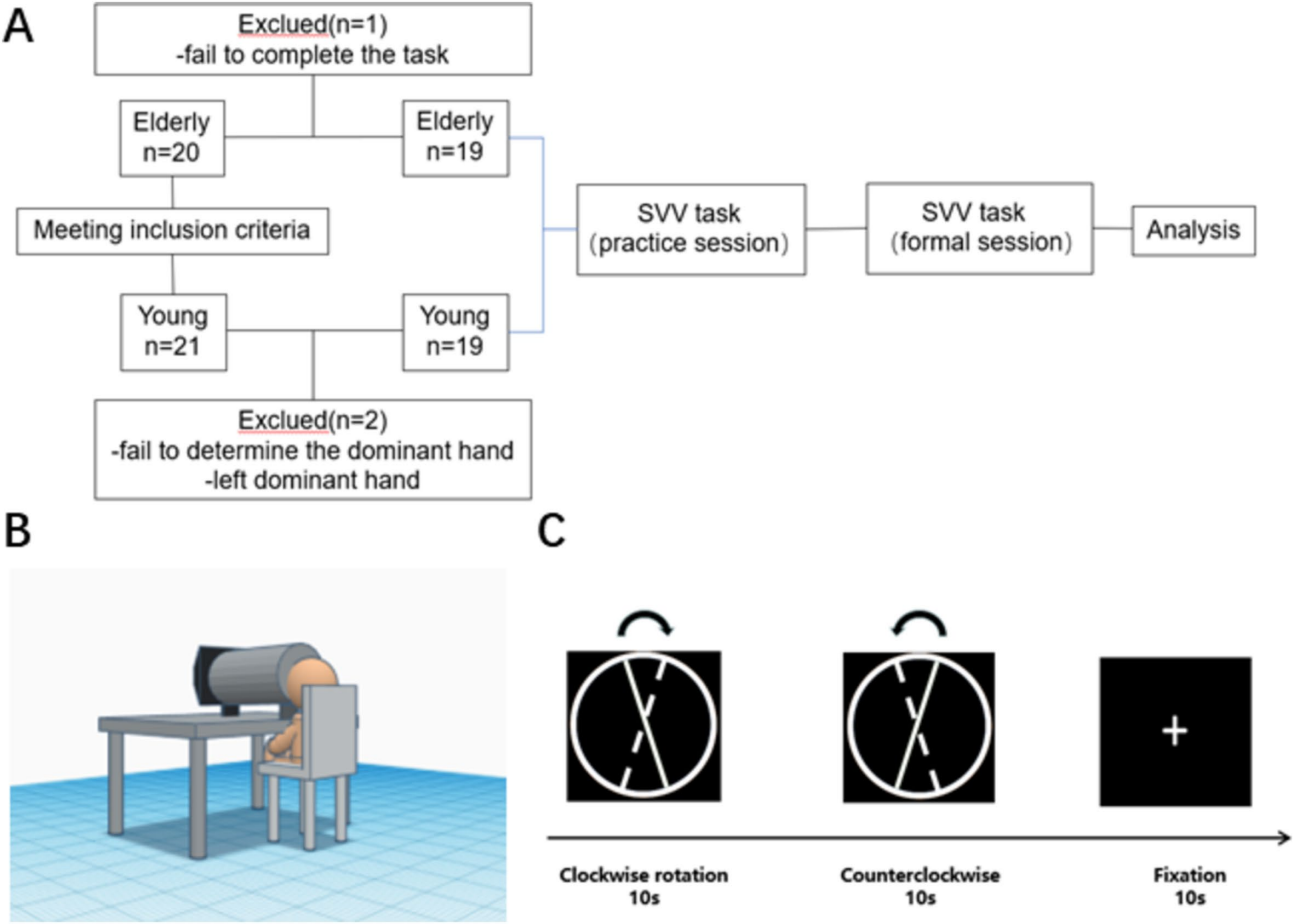
Study design. **(A)** Flow chart of the study. **(B)** Illustration of experiment setup. **(C)** Flow diagram of SVV test (The solid line represents the initial position, the dashed line represents the final position and the arrow represents the direction of rotation).

21 healthy young adults and 20 healthy old adults were recruited from the School of Rehabilitation Medicine and the First Affiliated Hospital of Nanjing Medical University. Two young people who could not determine the dominant hand or the dominant hand was not the right hand, and one older person who did not cooperate to complete the task was excluded. Finally, nineteen healthy young individuals, consisting of 6 males and 13 females, with an average age of 22.30 ± 2.05 years, and nineteen healthy older individuals, consisting of 10 males and 9 females with an age of 68.37 ± 4.67 years, were included in the trial. Informed consent was obtained from all participants (Figure 1A).

Inclusion criteria: ① Confirmation of right-sided dominance for both the dominant hand and the dominant foot using the Edinburgh Handedness Questionnaire (Oldfield, 1971) and the test of kick a ball (van Melick et al., 2017); ② Corrected visual acuity of 4.6 and above; ③ Age range of 22–30 years in the young and 60–75 years in the older people ④ Normal vestibular function, absence of any motion sicknesses symptoms such as vertigo, dizziness, or motion-induced nausea.

Exclusion criteria: ① The occurrence of diseases involving the vestibular system, skeletal muscular system, vision-related diseases, or damage to the central nervous system; ② The consumption of alcohol or drugs affecting balance-related factors in the last 48 h; ③ Excessive movements during the experiment, or another inability to cooperate in completing the test.

### 2.2 Study design

The ethics committee of The First Affiliated Hospital of Nanjing Medical University approved the experimental procedures (2023-SR-283).

#### 2.2.1 SVV task paradigm

Considering that the assessment position can affect the subject’s vertical perception condition and the acquisition of fNIRS data, the SVV task was performed based on a block design using Eprime 3.0 software, according to Piscicelli and Perennou (2017) for the normalization of SVV measurement paradigm after stroke. Additionally, considering the potential interference from the peripheral environment during testing, we implemented a bucket approach, similar to the bucket test (Zwergal et al., 2009), to control the field of view and prevent visual frame referencing. The experimental setup included a bucket with a diameter of 24 cm and a depth of 40.5 cm, positioned between the participant’s face and the display screen. Chairs of different heights were provided to ensure that the participant was at the proper level with the bucket and screen. The height of the bucket was also adjustable to accommodate different participants. Participants were instructed to place their faces inside the bucket and view the screen through it. The position of the bucket center was adjusted to align with the center of the video presented during the experimental procedure. The experimental setup is illustrated in Figure 1B.

The following standardized conditions for SVV assessment were carried out: ① The subject was in a seated position with the head and body in a vertical position (Piscicelli et al., 2016). Both hands were naturally placed on the table, and key-based decision-making was executed using the dominant hand; ② A height-adjustable chin-holding tool was used to keep the head and trunk in a vertical position at all times; ③ The number of trials was 6 blocks.

With a vertical position of 0° (using a heavy hammer line to confirm the position of gravity before starting the test) and a clockwise direction as the positive direction, the line segment was placed in a circular bottom background to avoid visual frame reference (Stapel and Medendorp, 2021). The display has a resolution of 1920*1080 and a screen size of 21.45 inches. The initial position of the line segment was −10°, and the line segment was rotated uniformly and slowly at 2°/s to the other side (continuous rotation at a frame rate of 24 frames/s). After the line segment was rotated clockwise to +10°, the direction of rotation was changed to counterclockwise rotation and again shifted to the initial −10° position at 2°/s (The stimulus line continues to move to the endpoint after the spacebar has been pressed). During the rotation of the line, the patient pressed the space bar, with the right hand, to confirm when he/she thought the line had reached the vertical position.

After two vertical perception tasks, there would be a 10-s resting period in which the subject was asked to gaze at the white cross on a black background without any other action. In this study, each block consists of one clockwise rotation, one counterclockwise rotation, and a 10-s annotation period, for a total of six blocks (Figure 1C). The SVV results were accurate to 0.1°. The keyboard was placed on the dominant side of the participant to facilitate responding, with no feedback provided after a key press. The E-Prime program recorded the keypresses.

SVV orientation (accuracy) is often obtained by averaging values from even-numbered measurements of vertical perception, reflecting the accuracy of the subject’s perception of gravity; SVV uncertainty (stability) is acquired by calculating the standard deviation after even-numbered measurements frequently, reflecting the speculative nature of the subject’s judgment of gravity perception, and the former is considered to be a relatively more reliable indicator (Piscicelli et al., 2015; Day et al., 2022).

This study correlates SVV metrics with brain activation, seeking to establish a bridge between imaging and behavior. At the same time, to make the relevant data calculation more clinically meaningful, we have optimized the SVV-related data calculation method. For the original SVV value, we take its absolute value to represent the deviation distance from the absolute 0° line for each measurement, and then obtain the modified SVV orientation and SVV uncertainty. The smaller the SVV metrics in this case, the higher the accuracy and stability of vertical perception.


$$\mathrm{modified}\;SVV\;\mathrm{orientation}=\mathrm{AVERAGE}\left(\mathrm{ABS}\left(SVV\right)\right)$$

$$\mathrm{modified}\;SVV\;\mathrm{uncertainty}=\mathrm{STDEV}\left(\mathrm{ABS}\left(SVV\right)\right)$$

SVV: The values of SVV measurement every time, 12 times total, ABS: Returns the absolute value of a given value; AVERAGE: Returns the arithmetic average of a given value; STDEV: Returns the standard deviation of a given sample.

#### 2.2.2 fNIRS

**Figure 2:**
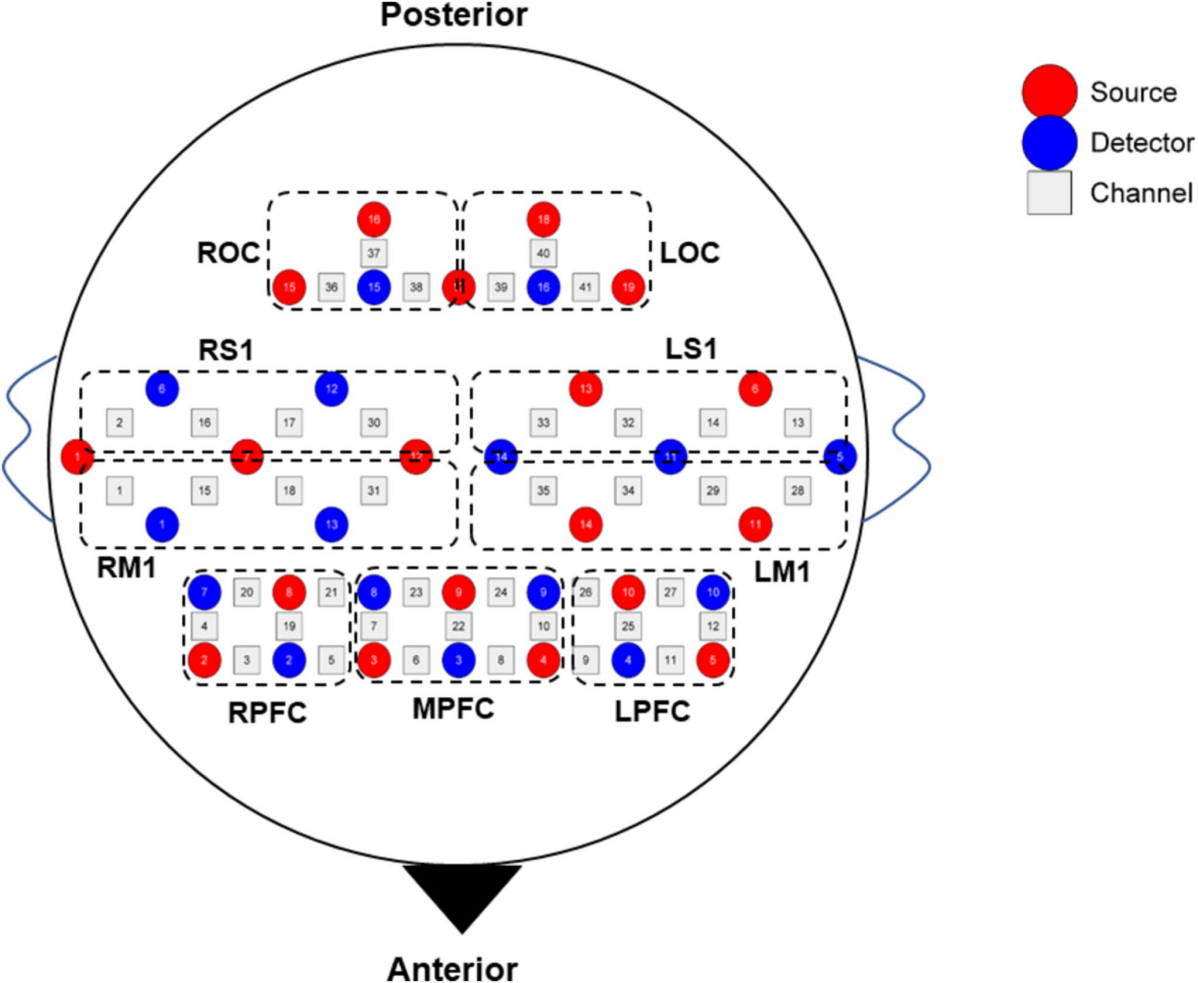
Diagram of channel layout. LPFC, left prefrontal cortex; MPFC, middle prefrontal cortex; RPFC, right prefrontal cortex; LM1, left primary motor cortex; RM1, right primary motor cortex; LS1, left primary sensory cortex; RS1, right primary sensory cortex; LOC, left occipital cortex; ROC, right occipital cortex.

The fNIRS data were collected simultaneously while subjects were performing the SVV task. The fNIRS device employed was a 41-channel NirSmart (NirSmart−3000A, Danyang Huichuang Medical Equipment Co., Ltd., China). The sampling frequency was 11 Hz. HbO and HbR concentrations were measured using continuous wave (CW) light-emitting diodes at 730 nm and 850 nm. The channel montage was arranged on a custom-designed head cap based on the 10/20 international standard system and the region of interest (ROI) related arrangement was designed concerning the study by Sui et al. (2022) (Figure 2). The spatial coordinates of the corresponding brain regions were confirmed using a standard head model with the Patriot localizer (Polhemus, USA). The corresponding brain regions were identified based on MNI and AAL coordinates, and the probability that each channel corresponded to the brain region was listed in Supplementary materials S1.

The test was conducted in a controlled environment with minimal light and noise interference. Before the test, the operator positioned an fNIRS cap on the subject’s head, aligning it with the Cz point at the intersection of the anterior line of the two ear screens and the nasal root-posterior occipital ridge. Signal channels were adjusted to ensure optimal signal transmission across all channels. After signal stabilization, the subject has to rest for 5 min before initiating the test. To minimize the impact of motion artifacts and other factors on the fNIRS signal, participants were instructed to avoid unnecessary movements during the fNIRS recording. Any additional movements or verbal expressions unrelated to the task were considered invalid for data analysis. Each subject received one practice session, which contained one trial, before the formal task.

#### 2.2.3 Data and statistical analysis

Statistical analysis was performed using IBM SPSS Statistics software (v25, IBM Corp, Armonk, NY, USA). The normality of the data was assessed using the Shapiro–Wilk test. Independent sample *T*-tests were conducted to compare SVV metrics between the young and the older individuals. Paired *T*-tests were used to compare SVV values between clockwise and counterclockwise rotations. The Pearson correlations were calculated between HbO concentration and SVV.

The HbO concentration signal was chosen as the primary indicator due to its sensitivity to changes in blood flow (Huppert et al., 2006). The fNIRS data preprocessing was conducted using Homer2 (Huppert et al., 2009) in Matlab 2013b (MathWorks, USA). The following steps were performed: (1) Conversion of light intensity to optical density. (2) Detection and correction of channel-by-channel motion artifacts using wavelet transform (parameters set as tMotion = 0.5, tMask = 1.0, STDEVthresh = 20.0, AMPthresh = 5.0, kurt = 1.5) (Molavi and Dumont, 2012). (3) Bandpass filtering of the signal within the frequency range of 0.01–0.08 Hz. (4) Conversion of optical density to blood oxygen concentration based on the modified Beer–Lambert law. (5) Additional correction for motion artifacts (Cui et al., 2010). Finally, a block averaging analysis was performed to estimate the hemodynamic response function (HRF) for each task.

Each block consists of a 20-s task period and a 10-s rest period. After averaging the six blocks, the mean HbO concentration from 5–20 s and 20–30 s is calculated. The difference between the task period and the rest period represents the relative change in HbO concentration during the task. This is expressed by the formula:


$$\mathrm{Diff}\;\left[HbO2\right]=\mathrm{Mean}\;\left[HbO2\right]\;5–20s-\mathrm{Mean}\;\left[HbO2\right]\;20–30\;s$$

The NIRS-SPM was used to detect brain area activation (Ye et al., 2009). The one-sample *t*-test was used to detect the activation of each channel with false discovery rate (FDR) correction for multiple comparisons. The BrainNet Viewer (Xia et al., 2013) was utilized for visualizing the channel montage and activation mapping in the brain cortex. Two therapists, each having over one year of experience in fNIRS operation and analysis, collaborated in conducting the test and performing signal acquisition and analysis.

According to the previous study, the results of the block average are considered more suitable for brain functional lateralization calculation (Borrell et al., 2023). Several approaches for brain functional lateralization in fNIRS have been applied, taking cues from the lateralization methods used in fMRI. This study employed three approaches for computation, as shown below (Seghier, 2008; Borrell et al., 2023).

Method①


$$\begin{array}{l} LI=\left(\mathrm{ABS}\left(\left[HbO\right]L\right)-\mathrm{ABS}\left(\left[HbO\right]R\right)\right)/\\ \;\left(\mathrm{ABS}\left(\left[HbO\right]L\right)+\mathrm{ABS}\left(\left[HbO\right]R\right)\right) \end{array}$$

Method②


$$LI=\left(\left[HbO\right]L-\left[HbO\right]R\right)/\left(\mathrm{ABS}\left(\left[HbO\right]L\right)+\mathrm{ABS}\left(\left[HbO\right]R\right)\right)$$

Method③


$$LI=\left(\left[HbO\right]L-\left[HbO\right]R\right)/\left(\left[HbO\right]L+\left[HbO\right]R\right)$$

(ABS represents the absolute value taken for the relevant data; [HbO]represents the concentration of HbO; [HbO]L represents the HbO concentration of the region in the left brain; [HbO]R represents the HbO concentration of the region in the right brain).

In this study, a group-level criterion of HbO concentration, LI ≥ 0.1 was used to indicate left lateralization, while LI ≤ −0.1 was considered as right lateralization. The range between −0.1 and 0.1 was interpreted as bilateral dominance (Vernooij et al., 2007).

## 3 Results

### 3.1 SVV orientation and uncertainty

To reduce the effect of the starting position of the line on the SVV estimate (Baccini et al., 2014), we calculated the SVV result based on 12 times total, including clockwise and counterclockwise rotation. In this trial, the participants exhibited an SVV orientation of −0.21 ± 0.88° and an SVV uncertainty of 0.88 ± 0.30° in the young, 0.59 ± 1.20° and 1.84 ± 1.80° in the older.

**Figure 3:**
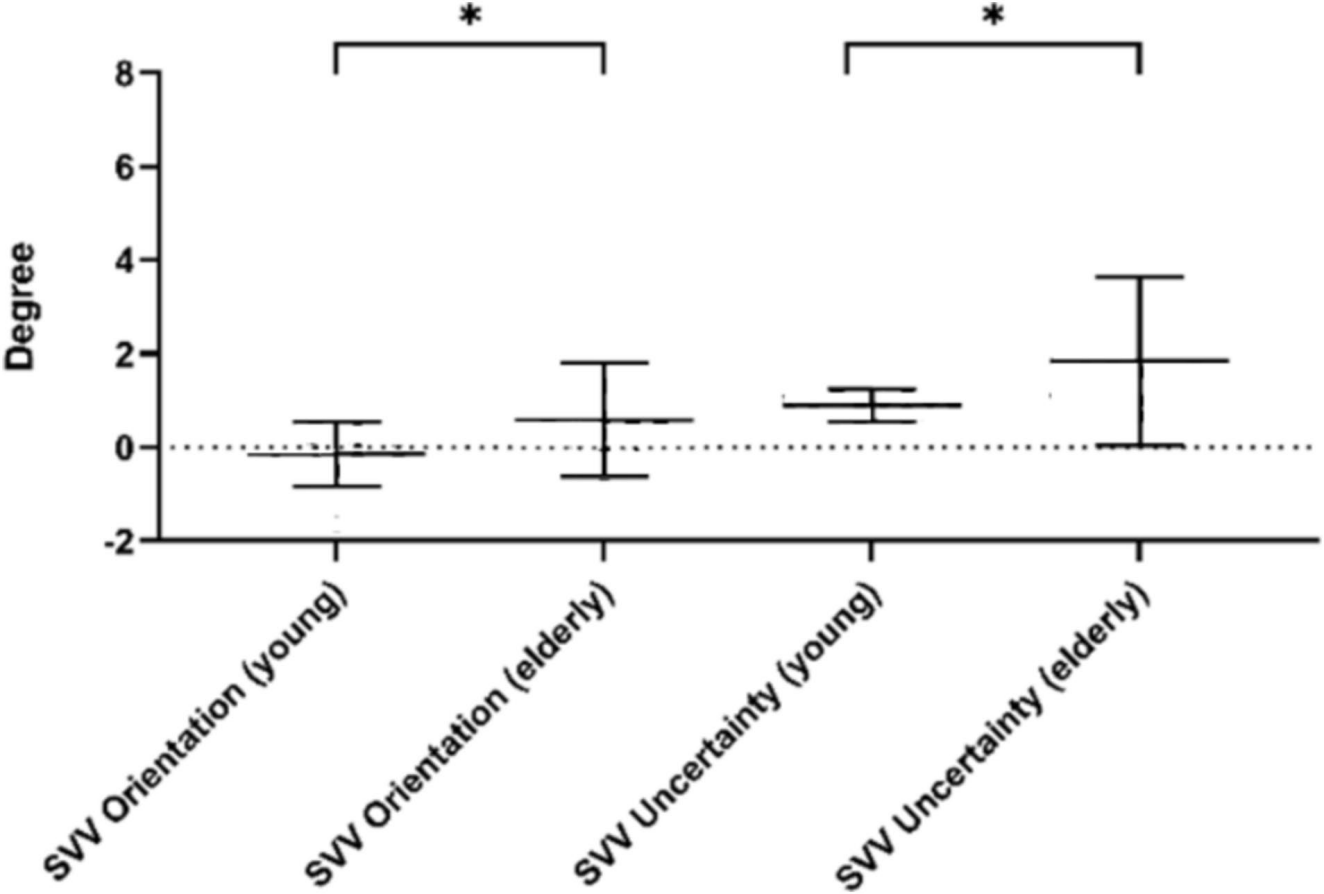
SVV results of the young and the elder subjects. *p < 0.05.

The SVV orientation of young people was significantly different from that of the older people (*t* = 2.294, *p* = 0.028), which was closer to the complete vertical position. It means that young people have a more accurate perception of SVV. Similarly, the SVV uncertainty of young people is significantly different from that of older people (*t* = 2.215, *p* = 0.033), and they have lower scores, which means that young people have more certainty about the absolute vertical position, rather than more uncertainty of older people. Detailed information is shown in Figure 3.

In SVV metrics, including SVV uncertainty and SVV orientation, no significant difference was found between the clockwise and counterclockwise rotation trials (*t* = 0.260, *p* = 0.796), both among the older and young people.

### 3.2 Block averaging

After block averaging and FDR correction, channels 2 and 35 exhibited activation in two groups. These channels corresponded to brain regions, specifically the right SMG and the left superior frontal gyrus of the dorsolateral (SFGdor). Moreover, additional channels 9, 14, 16, 17, 18, 30, 31, 32, 33, and 37 exhibited activation in the older individuals. The relative information about the results is shown in Table 1.

**Table 1:** Significantly activated channels in the young and the older individuals.

Population	Channel	MNI	AAL*	The most probability corresponding brain area	Concentration of HbO		
		X Y Z			*t*	*p (raw)*	*p (FDR)*
Young	CH2 (S1-D6)	63 –30 52	SupraMarginal_R	54.511%	3.619	0.002	0.040
	CH35 (S14-D14)	–30 1 68	Frontal_Sup_L	61.888%	3.863	0.001	0.040
Older	CH2 (S1-D6)	63 –30 52	SupraMarginal_R	54.511%	3.047	0.007	0.026
	CH9 (S4-D4)	−32 65 –11	Frontal_Mid_Orb_L	82.510%	2.835	0.011	0.038
	CH14 (S6-D11)	−52 –25 62	Postcentral_L	96.800%	3.108	0.006	0.025
	CH16 (S7-D6)	52 –28 63	Postcentral_R	72.941%	3.429	0.003	0.025
	CH17 (S7-D12)	41 –27 70	Postcentral_R	52.708%	3.342	0.004	0.025
	CH18 (S7-D13)	42 –4 65	Frontal_Mid_R	40.441%	4.640	<0.001	0.005
	CH30 (S12-D12)	31 –26 74	Precentral_R	68.404%	3.099	0.006	0.025
	CH31 (S12-D13)	29 –4 70	Frontal_Sup_R	88.849%	4.056	0.001	0.010
	CH32 (S13-D11)	−41 –24 69	Postcentral_L	53.261%	3.171	0.005	0.025
	CH33 (S13-D14)	−31 –21 74	Precentral_L	73.244%	4.528	<0.001	0.005
	CH35 (S14-D14)	−30 1 68	Frontal_Sup_L	61.888%	3.324	0.004	0.025
	CH37 (S16-D15)	22 –101 19	Occipital_Sup_R	89.855%	3.129	0.006	0.025

^*^AAL, The listed are the most likely corresponding brain areas in the AAL template.

**Figure 4:**
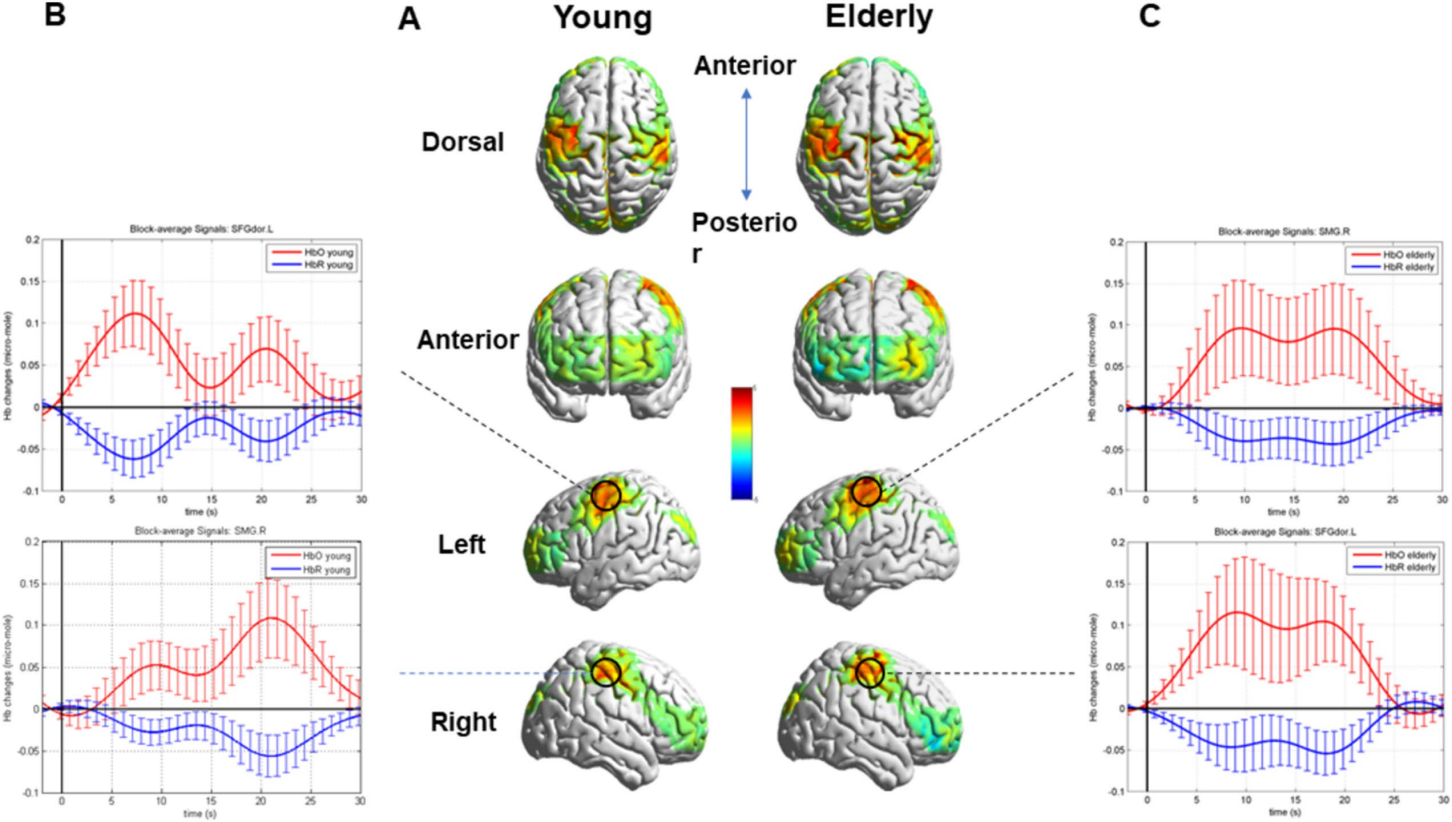
Whole brain activation during the SVV task in two groups and the HRF of the Activated Channels 2 and 35. **(A)** Overall brain activation of the young and the older. **(B)** The HRF of the Activated Channels 2 and 35 in the young during the SVV task. (C) The HRF of the Activated Channels 2 and 35 in the older during the SVV task.

The task’s HRF of the co-activated channels, channels 2 and 35, and the overall brain activation are visualized in Figure 4.

### 3.3 Relation between the SVV metrics and the HbO concentration

In the young population, CH2, CH5, CH16, RM1, and RS1 were significantly negatively correlated with modified SVV orientation. CH26 was negatively correlated with modified SVV orientation and modified SVV uncertainty. That is, the lower the activation of these brain regions, the higher the modified SVV values, and the worse the SVV performance. CH12 was positively correlated with modified SVV orientation and modified SVV uncertainty. That is, the lower the CH12 activation, the lower the modified SVV values, and the better the SVV performance.

Differently, in the older individuals, CH7, RM1, and LPFC were significantly negatively correlated with modified SVV orientation. CH12, CH18, and CH26 were significantly negatively correlated with modified SVV orientation and modified SVV uncertainty. That is, the lower the activation of these brain regions, the higher the modified SVV values, and the worse the SVV performance. No brain regions showed a positive correlation with SVV metrics.

**Figure 5:**
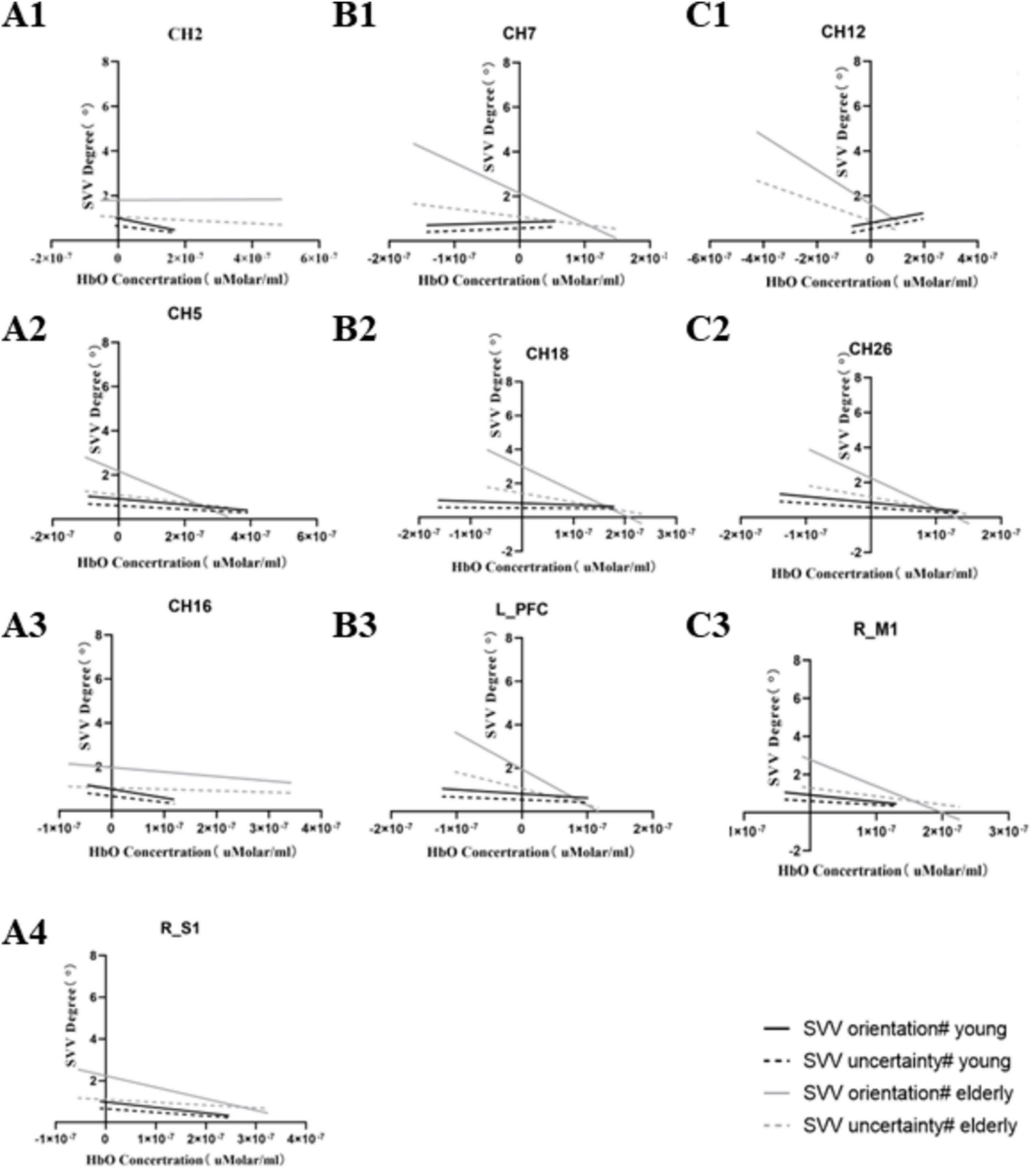
Significantly correlated channels and ROI with SVV metrics. Series A represents the significantly related channels only in the young, series B represents the significantly related channels only in the older, and series C represents the significantly co-related channels in both groups.

The correlation with a significant meaning, in the two populations, is presented in Tables 2, 3. The results of linear regression of relevant data are presented in Figure 5.

**Table 2:** Correlation between brain functional imaging and SVV metrics in the young.

Channels/ROIs	2	5	12	16	26	LM1	RM1	LS1	RS1	LPFC	RPFC	LOC	ROC
SVV													
AAL Coordinate System	Right supramarginal gyrus (SMG.R)	Right Middle frontal gyrus, orbital part (ORBmid.R)	Left Inferior frontal gyrus, triangular part (IFGtriang.L)	Right postcentral gyrus (PoCG.R)	Left middle frontal gyrus (MFG.L)	/	/	/	/	/	/	/	/
SVV orientation#	−0.573*	−0.612*	0.558*	−0.607*	−0.837**	−0.303	−0.648*	−0.173	−0.602*	−0.400	−0.513	−0.023	0.360
SVV uncertainty#	−0.364	−0.441	0.694**	−0.509	−0.680*	0.315	0.017	0.571	0.029	0.175	0.073	0.941	0.227

The Pearson correlation coefficients between the HbO of brain regions and SVV metrics (for all ROI and channels that have a significant correlation with at least one SVV metric); *Significant correlation at the 0.05 level (two-tailed); ** Significant correlation at the 0.01 level (two-tailed); SVV orientation# represents modified SVV orientation, SVV uncertainty# represents modified SVV uncertainty.

**Table 3:** Correlation between brain functional imaging and SVV metrics in the older.

Channels/ROIs	7	12	18	26	LM1	RM1	LS1	RS1	LPFC	RPFC	LOC	ROC
SVV												
AAL Coordinate System	Right superior frontal gyrus, dorsolateral	Left inferior frontal gyrus, triangular part	Right middle frontal gyrus	Left middle frontal gyrus	/	/	/	/	/	/	/	/
SVV orientation#	−0.677**	−0.473*	−0.636**	−0.572*	−0.258	−0.503*	−0.148	−0.251	−0.478*	−0.305	−0.137	−0.264
SVV uncertainty#	−0.439	−0.643**	−0.568*	−0.548*	0.287	0.028	0.545	0.300	0.039	0.205	0.577	0.274

The Pearson correlation coefficients between the HbO of brain regions and SVV metrics (for all ROI and channels that have a significant correlation with at least one SVV metric); *Significant correlation at the 0.05 level (two-tailed); **Significant correlation at the 0.01 level (two-tailed); SVV orientation# represents modified SVV orientation, SVV uncertainty# represents modified SVV uncertainty.

### 3.4 Laterality index (LI)

The results of brain functional lateralization which were calculated according to three methods are presented in Table 4. The results demonstrate that, in the young, the Sensory Cortex and the Dorsolateral Prefrontal Cortex were right lateralization, and the Motor Cortex and the Occipital Cortex were left lateralization. However, in the older individuals, the Sensory Cortex, the Dorsolateral Prefrontal Cortex, and the Motor Cortex were bilateral dominant and the Occipital Cortex was right lateralization.

**Table 4:** Laterality index of ROI regions.

ROI (Channels)	Age	HbO concentration_left	HbO concentration_right	LI①	LI②	LI③	Lateralization
Motor Cortex (Left:28,29,34,35;Right:1,15,18,31)	Young	6.08036E-08	4.02734E-08	0.203	0.203	0.203	Left
	Older	8.04538E-08	6.93342E-08	0.074	0.074	0.074	Bilateral
Sensory Cortex (Left:13,14,32,33;Right:2,16,17,30)	Young	3.91357E-08	5.06995E-08	−0.129	−0.129	−0.129	Right
	Older	8.38481E-08	7.63697E-08	0.047	0.047	0.047	Bilateral
Dorsolateral Prefrontal Cortex (Left:CH9,11,12,25,26,27;Right:CH3,4,5,19,20,21)	Young	−1.18669E-09	1.45356E-08	−0.849	−1.000	−1.178	Right
	Older	8.48293E-09	7.0837E-09	0.090	0.090	0.090	Bilateral
Occipital Cortex (Left:39,40,41 Right:36,37,38)	Young	3.18E-08	1.49E-08	0.361	0.361	0.361	Left
	Older	2.08342E-09	3.46532E-08	−0.887	−0.887	−0.887	Right

The population level values of HbO concentration were taken for LI calculation; LI ①, LL ②, and LI ③ represent the results obtained using Method ①, Method ②, and Method ③, respectively.

## 4 Discussion

### 4.1 SVV orientation and uncertainty

The SVV orientation obtained from the 19 healthy young adults and 19 healthy old people in the present study was in the normal range (Perennou et al., 2008). Moreover, no significant difference was found between clockwise and counterclockwise rotations.

This experiment also verifies the previous studies, confirming that the young showed a more accurate perception of SVV direction and better stability (Cakrt et al., 2016). Indeed, increasing age can cause more uncertainty and judgment instability for SVV. Aging is confirmed to be one of the factors that influence the SVV task (Cakrt et al., 2016). With increasing age, there is a decrease in reliance on vestibular information while the reliance on visual input is increased. The contribution of vestibular and visual cues to vertical perception should be reevaluated and adjusted based on age (Alberts et al., 2019). In this study, we chose the use of absolute values for SVV-related indicators, which seems to supplement the assessment of an individual’s SVV perception ability to some extent (Piscicelli and Perennou, 2017).

### 4.2 Cortical activation of the SVV task

The block averaging results of HbO concentration, corrected FDR, demonstrated that the right SMG and left SFGdor, were activated in the young and the older. However, in the older individuals, more brain areas were activated, especially the postcentral gyrus (PoCG) of both sides, the right middle frontal gyrus. Moreover, the left middle orbitofrontal gyrus, precentral gyrus of both sides, and right superior frontal gyrus were also activated in the older.

The cerebral cortex contains widespread functional areas that receive vestibular-related signals, playing an essential role in various cognitive processes such as visuospatial cognition, spatial navigation, orientation, and self-motor perception. The vestibular cortex, containing the parietoinsular vestibular cortex and posterior insular cortex, represents key components of the vestibular cortical network (Frank and Greenlee, 2018; Indovina et al., 2020) and subjective visual vertical perception (Jafari et al., 2023). The right temporoparietal junction (rTPJ) is crucial for integrating sensory information from multiple systems, including the vestibular and somatic systems. The primary function of rTPJ is to maintain an internal representation of verticality by combining these sensory inputs (Fiori et al., 2015). Kheradmand et al. reported that the SVV collected from 8 subjects was tilted to the opposite direction of the initial head position (right: 3.6°, left: 2.7°) after receiving a continuous theta burst stimulation (cTBS), a kind of transcranial magnetic stimulation (TMS), applied to the right SMG region, while no significant tilt was found after applying cTBS to the nearby areas (Kheradmand et al., 2015). The intervention at the corresponding site not only demonstrated target location specificity but also task specificity. After the same pattern of TMS on the intraparietal sulcus nearest the dorsal tip of the right SMG (repetitive trains of 6 pulses, a frequency of 10 Hz, and an intensity of 110% of the active motor threshold), improvements of SVV bias occurred, but there was no significant change in vertical landmark control task (Willacker et al., 2019). This indicates the significant role of the SMG in integrating information from diverse sensory modalities to achieve the precise perception of an upright position or vertical orientation. Damage or lesions in the vestibular cortex and the pathway connecting the vestibular cortex and vestibular nuclei can disrupt the functional network involved in vertical perception. Consequently, this disruption can lead to impaired vertical perception (Brandt et al., 1994; Yeo et al., 2018). The present study also confirmed by fNIRS that the participation of the vestibular cortex, specifically the right SMG as the primary core area, is necessary for the SVV task. The SFG, parcellated into parts of the anteromedial, the dorsolateral, and the posterior, plays an important role in a variety of cognitive functions, especially working memory and executive functioning. Lateral and posterior parts of the left SFG are key components of the working memory network and are also deeply involved in the task of spatial orientation (du Boisgueheneuc et al., 2006; Li et al., 2013). Moreover, In addition, SFG is typically associated with an individual’s executive functions, and its abnormalities can lead to a certain degree of executive dysfunction (Jones and Graff-Radford, 2021).

Compared with the young, the older show more brain areas activated during the task, such a phenomenon can contribute to the effects of aging on cognition. Aging may lead to the need for more brain regions to perform certain tasks, and fNIRS can finish such detection (Bierre et al., 2017). This study also confirms this idea to some extent. The PoCG contains the primary somatosensory cortex, which is responsible for proprioception (DiGuiseppi and Tadi, 2023). A study found that in the primary somatosensory cortex of rats, neuronal firing patterns resembling location cells, head directional cells, and border cells were observed (Long and Zhang, 2021). These findings suggest the presence of a spatial navigation system within the somatosensory area, which may explain why older adults need more activation from this region to complete the SVV task. The right MFG, located at the intersection of the dorsal and ventral attention networks, is implicated in various attention-related functions, including selective and sustained attention (Japee et al., 2015). It plays a critical role in maintaining the integrity of the attention network, specifically involved in redirecting attention (Song et al., 2019). The theory of neural compensation suggests that elder participants recruit additional neural resources, compared with the young, to maintain cognitive task performance (Steffener et al., 2009). When completing the cognitive task of SVV, compared with young people, older people also recruited more resources in brain regions such as bilateral PoCG, right MFG, left middle orbitofrontal gyrus, precentral gyrus of both sides and right superior frontal gyrus, which is in line with neural compensation theory.

### 4.3 The correlation between the brain image and behavior

fNIRS can finish the assessment task of age-related decline of neurovascular coupling responses (Csipo et al., 2019). In this study, at the levels of channels and ROIs, we conducted a correlation analysis between the brain image and behavior. Among the channels or ROIs that showed significant correlations, modified SVV orientation and SVV uncertainty showed negative correlations in most cases (except for CH12 in the young). Higher activation in brain regions meant lower SVV values, that is, a more accurate and stable judgment of SVV.

In spatial memory function, linear or quadratic relationships were found between working memory load and PFC activity. Healthy older adults have higher levels of PFC activity than healthy adults, behavioral outcomes were worse. The results of the present study support this situation to some extent, because the excessive activation of relevant brain regions during the performance of specific tasks in the older may be a compensatory strategy. It has been suggested that more challenging visual–spatial tasks have greater potential to reveal more significant deficits in brain function (Butters et al., 2023). To some extent, this study supports that SVV can be included in the test suite of visual–spatial tasks.

### 4.4 Brain functional lateralization

Brain functional lateralization is considered a possible biomarker for dementia, reflecting the recruitment of contralateral resources to finish cognitive tasks (Yeung et al., 2016; Liu et al., 2018; Butters et al., 2023). When evaluating brain functional lateralization using fNIRS, it appears reasonable to encourage the use of block averaging analysis rather than GLM (Borrell et al., 2023). The results indicate predominant activation in the right hemisphere during the SVV task in the young, which aligns with the right hemispheric functional lateralization observed in the vestibular system for multisensory orientation in right-handers (Dieterich and Brandt, 2018). However, in the older individuals, it seems that bilateral dominance is the mainstream. Brain asymmetry is associated with behavioral and cognitive abilities and can change with physiological processes such as disease and aging (Hao et al., 2023). According to the HAROLD Model, the asymmetry of brain function in the older is reduced, and this process may have a physiological significance to play a compensatory function (Cabeza, 2002). This may explain, to some extent, the situation in this study in which older people present a bilateral advantage.

Due to the influence of handedness on functional lateralization (Szaflarski et al., 2002), all participants in this experiment were right-handed. However, considering the response requirements of the experimental paradigm, which involved using the right hand, finger tapping itself can have an impact on brain activation (Anwar et al., 2016; de Rond et al., 2023). For this reason, in this study, the motor cortex showed left-sided lateralization in the young. Subsequent research should propose more effective approaches to address this issue. An optimized and corrective approach needs to be proposed for the assessment of hemispheric lateralization conducted during the tasks, which need to use a single hand to make the response.

### 4.5 Restriction

In this experiment, the SVV task required participants to press a button to make a choice when the rotating line reached a vertical position. However, due to the effects of aging, older participants would require more time to make decisions. Consequently, the reaction time of the older population becomes a potential factor affecting the SVV indicator. Nevertheless, considering the research by Abdul Razzak et al. (2020), which utilized an active paradigm of SVV—where participants actively adjusted the line segment to what they perceived as the vertical position—demonstrated differences in SVV between young and older individuals. In this study, we employed a passive paradigm of SVV that is more suitable for fNIRS experimental design, focusing on exploring the differences in brain functional activation between two populations. Future research will need to strike a balance between seeking the applicability of the fNIRS paradigm and the characteristics of the participant population. Moreover, the conclusions of this study are based on a relatively small sample size and exhibit quite large variation. Future research needs to expand the sample size to obtain more generalizable conclusions.

## 5 Conclusion

The right SMG and the left SFGdor are likely the brain regions associated with SVV. With a huge probability, higher activation in specific brain regions means better performance on SVV tasks, except for left IFGtriang in the young. Due to age, the older individuals show bilateral recruitment of more brain areas and brain functional lateralization is bilateral when performing SVV. Additionally, in exploring the behavioral and mechanistic changes of aging, SVV is a good visuospatial task. Future research should focus on investigating the SVV characteristics in more different age groups and populations with various diseases. Additionally, it is imperative to further explore the aforementioned brain regions as potential targets for therapeutic interventions aimed at ameliorating SVV in patients and enhancing their overall functionality.

## Data Availability

The raw data supporting the conclusions of this article will be made available by the authors, without undue reservation.
