# Supplementary material for: Age-related differences of subjective visual vertical perception in adults—a functional near-infrared spectroscopy study
Source: Front Aging Neurosci. 2025 Jan 7;16:1449455. doi: 10.3389/fnagi.2024.1449455 (PMC11752877; doi:10.3389/fnagi.2024.1449455)
Supplement: Supplementary file 3 [file Table_3.DOCX]

Supplementary Material

Age-related differences of subjective visual vertical perception in adults-a functional near-infrared spectroscopy (fNIRS) study

**Jun Lu 1,2†, Xiang Gong 2†, Meng-Huan Wang 3†, Ruo-Xin Zhao 4, Yu-Chen Wang 5, Ying-Ying Shen 6, Rong Cao 1,* and Guang-Xu Xu 1, 2,***

**1 Department of Rehabilitation Medicine Center, The First Affiliated Hospital of Nanjing Medical University, Nanjing, Jiangsu, China**

**2 School of Rehabilitation Medicine, Nanjing Medical University, Nanjing, Jiangsu, China**

**3 School of Chinese Language and Literature, Nanjing Normal University, Nanjing, Jiangsu, China**

**4 Office of Clinical Trial Institution, School of Medicine, Nanjing Zhongda Hospital, Southeast University, Nanjing, Jiangsu, China**

**5 Department of Health Promotion Center, The First Affiliated Hospital with Nanjing Medical University, Nanjing, Jiangsu, China**

**6 School of Sport and Health, Guangzhou Sport University, Guangzhou, Guangdong, China.**

*** Correspondence:**Guang-Xu Xu
xuguangxu@njmu.edu.cn

# Supplementary 1 (S1)

| **The MNI coordinates and anatomical labels corresponding to the channels** | | | | | | |
| --- | --- | --- | --- | --- | --- | --- |
| **Channel number (S-D)** | **MNI** | | | **AAL** | **Proportion** |  |
|  | **x** | **y** | **z** |  |  |  |
| CH1 (S1-D1) | 60 | -5 | 47 | Precentral_R | 0.51504 |  |
|  |  |  |  | Frontal_Mid_R | 0.078947 |  |
|  |  |  |  | Postcentral_R | 0.40602 |  |
| CH2 (S1-D6) | 63 | -30 | 52 | Postcentral_R | 0.067669 |  |
|  |  |  |  | Parietal_Inf_R | 0.38722 |  |
|  |  |  |  | SupraMarginal_R | 0.54511 |  |
| CH3 (S2-D2) | 53 | 44 | -12 | Frontal_Mid_Orb_R | 0.11151 |  |
|  |  |  |  | Frontal_Inf_Orb_R | 0.88849 |  |
| CH4 (S2-D7) | 59 | 31 | 3 | Frontal_Inf_Tri_R | 0.80192 |  |
|  |  |  |  | Frontal_Inf_Orb_R | 0.19808 |  |
| CH5 (S3-D2) | 38 | 64 | -12 | Frontal_Sup_Orb_R | 0.16016 |  |
|  |  |  |  | Frontal_Mid_Orb_R | 0.83984 |  |
| CH6 (S3-D3) | 14 | 71 | -12 | Frontal_Sup_Orb_R | 0.53512 |  |
|  |  |  |  | Frontal_Mid_Orb_R | 0.46488 |  |
| CH7 (S3-D8) | 28 | 69 | 2 | Frontal_Sup_R | 0.62295 |  |
|  |  |  |  | Frontal_Sup_Orb_R | 0.33443 |  |
|  |  |  |  | Frontal_Mid_R | 0.042623 |  |
| CH8 (S4-D3) | -10 | 71 | -11 | Frontal_Sup_Orb_L | 0.51195 |  |
|  |  |  |  | Frontal_Mid_Orb_L | 0.44027 |  |
|  |  |  |  | Rectus_L | 0.047782 |  |
| CH9 (S4-D4) | -32 | 65 | -11 | Frontal_Sup_L | 0.041825 |  |
|  |  |  |  | Frontal_Sup_Orb_L | 0.12928 |  |
|  |  |  |  | Frontal_Mid_Orb_L | 0.8251 |  |
|  |  |  |  | Frontal_Inf_Orb_L | 0.0038023 |  |
| CH10 (S4-D9) | -20 | 72 | 4 | Frontal_Sup_L | 0.7907 |  |
|  |  |  |  | Frontal_Sup_Orb_L | 0.10631 |  |
|  |  |  |  | Frontal_Mid_L | 0.013289 |  |
|  |  |  |  | Frontal_Sup_Medial_L | 0.053156 |  |
|  |  |  |  | Frontal_Mid_Orb_L | 0.036545 |  |
| CH11 (S5-D4) | -50 | 43 | -14 | Frontal_Mid_Orb_L | 0.029412 |  |
|  |  |  |  | Frontal_Mid_Orb_L | 0.029412 |  |
| CH12 (S5-D10) | -57 | 27 | 2 | Frontal_Inf_Oper_L | 0.0063898 |  |
|  |  |  |  | Frontal_Inf_Tri_L | 0.84026 |  |
|  |  |  |  | Frontal_Inf_Orb_L | 0.15335 |  |
| CH13 (S6-D5) | -61 | -27 | 50 | Postcentral_L | 0.13553 |  |
|  |  |  |  | Parietal_Inf_L | 0.48352 |  |
|  |  |  |  | SupraMarginal_L | 0.38095 |  |
| CH14 (S6-D11) | -52 | -25 | 62 | Postcentral_L | 0.968 |  |
|  |  |  |  | Parietal_Inf_L | 0.032 |  |
| CH15 (S7-D1) | 52 | -5 | 57 | Precentral_R | 0.46639 |  |
|  |  |  |  | Frontal_Mid_R | 0.47899 |  |
|  |  |  |  | Postcentral_R | 0.054622 |  |
| CH16 (S7-D6) | 52 | -27 | 63 | Precentral_R | 0.039216 |  |
|  |  |  |  | Postcentral_R | 0.72941 |  |
|  |  |  |  | Parietal_Sup_R | 0.098039 |  |
|  |  |  |  | Parietal_Inf_R | 0.13333 |  |
| CH17 (S7-D12) | 41 | -27 | 70 | Precentral_R | 0.47292 |  |
|  |  |  |  | Postcentral_R | 0.52708 |  |
| CH18 (S7-D13) | 42 | -4 | 65 | Precentral_R | 0.38235 |  |
|  |  |  |  | Frontal_Sup_R | 0.21324 |  |
|  |  |  |  | Frontal_Mid_R | 0.40441 |  |
| CH19 (S8-D2) | 47 | 56 | 1 | Frontal_Mid_R | 0.53488 |  |
|  |  |  |  | Frontal_Mid_Orb_R | 0.33721 |  |
|  |  |  |  | Frontal_Inf_Tri_R | 0.073643 |  |
|  |  |  |  | Frontal_Inf_Orb_R | 0.054264 |  |
| CH20 (S8-D7) | 53 | 42 | 20 | Frontal_Mid_R | 0.53309 |  |
|  |  |  |  | Frontal_Inf_Tri_R | 0.46691 |  |
| CH21 (S8-D8) | 38 | 60 | 18 | Frontal_Sup_R | 0.27642 |  |
|  |  |  |  | Frontal_Mid_R | 0.72358 |  |
| CH22 (S9-D3) | 5 | 72 | 3 | Frontal_Sup_R | 0.018987 |  |
|  |  |  |  | Frontal_Sup_Orb_R | 0.041139 |  |
|  |  |  |  | Frontal_Sup_Medial_L | 0.22785 |  |
|  |  |  |  | Frontal_Sup_Medial_R | 0.47468 |  |
|  |  |  |  | Frontal_Mid_Orb_L | 0.10759 |  |
|  |  |  |  | Frontal_Mid_Orb_R | 0.12975 |  |
| CH23 (S9-D8) | 17 | 70 | 21 | Frontal_Sup_R | 0.60825 |  |
|  |  |  |  | Frontal_Mid_R | 0.020619 |  |
|  |  |  |  | Frontal_Sup_Medial_R | 0.37113 |  |
| CH24 (S9-D9) | -11 | 70 | 20 | Frontal_Sup_L | 0.59028 |  |
|  |  |  |  | Frontal_Sup_Medial_L | 0.40972 |  |
| CH25 (S10-D4) | -45 | 55 | 2 | Frontal_Mid_L | 0.48846 |  |
|  |  |  |  | Frontal_Mid_Orb_L | 0.39231 |  |
|  |  |  |  | Frontal_Inf_Tri_L | 0.11538 |  |
|  |  |  |  | Frontal_Inf_Orb_L | 0.0038462 |  |
| CH26 (S10-D9) | -34 | 60 | 20 | Frontal_Sup_L | 0.27888 |  |
|  |  |  |  | Frontal_Mid_L | 0.72112 |  |
| CH27 (S10-D10) | -52 | 38 | 19 | Frontal_Mid_L | 0.23022 |  |
|  |  |  |  | Frontal_Inf_Tri_L | 0.76978 |  |
| CH28 (S11-D5) | -59 | -3 | 43 | Precentral_L | 0.36727 |  |
|  |  |  |  | Precentral_L | 0.36727 |  |
| CH29 (S11-D11) | -51 | -1 | 55 | Precentral_L | 0.728 |  |
|  |  |  |  | Frontal_Mid_L | 0.028 |  |
|  |  |  |  | Postcentral_L | 0.244 |  |
| CH30 (S12-D12) | 31 | -26 | 74 | Precentral_R | 0.68404 |  |
|  |  |  |  | Postcentral_R | 0.31596 |  |
| CH31 (S12-D13) | 29 | -4 | 70 | Precentral_R | 0.086331 |  |
|  |  |  |  | Frontal_Sup_R | 0.88849 |  |
|  |  |  |  | Frontal_Mid_R | 0.02518 |  |
| CH32 (S13-D11) | -41 | -24 | 69 | Precentral_L | 0.46739 |  |
|  |  |  |  | Postcentral_L | 0.53261 |  |
| CH33 (S13-D14) | -31 | -21 | 74 | Precentral_L | 0.73244 |  |
|  |  |  |  | Postcentral_L | 0.26756 |  |
| CH34 (S14-D11) | -40 | 1 | 63 | Precentral_L | 0.73308 |  |
|  |  |  |  | Frontal_Sup_L | 0.041353 |  |
|  |  |  |  | Frontal_Mid_L | 0.22556 |  |
| CH35 (S14-D14) | -30 | 1 | 68 | Precentral_L | 0.22378 |  |
|  |  |  |  | Frontal_Sup_L | 0.61888 |  |
|  |  |  |  | Frontal_Mid_L | 0.15734 |  |
| CH36 (S15-D15) | 31 | -89 | 35 | Occipital_Sup_R | 0.55882 |  |
|  |  |  |  | Occipital_Mid_R | 0.44118 |  |
| CH37 (S16-D15) | 22 | -101 | 19 | Cuneus_R | 0.07971 |  |
|  |  |  |  | Occipital_Sup_R | 0.89855 |  |
|  |  |  |  | Occipital_Mid_R | 0.021739 |  |
| CH38 (S17-D15) | 13 | -96 | 31 | Cuneus_L | 0.066176 |  |
|  |  |  |  | Cuneus_R | 0.21324 |  |
|  |  |  |  | Occipital_Sup_R | 0.72059 |  |
| CH39 (S17-D16) | -15 | -97 | 30 | Cuneus_L | 0.11913 |  |
|  |  |  |  | Occipital_Sup_L | 0.88087 |  |
| CH40 (S18-D16) | -25 | -101 | 17 | Occipital_Sup_L | 0.36727 |  |
|  |  |  |  | Occipital_Mid_L | 0.63273 |  |
| CH41 (S19-D16) | -35 | -90 | 31 | Occipital_Sup_L | 0.10256 |  |
|  |  |  |  | Occipital_Mid_L | 0.89744 |  |
